# Urate and Nonanoate Mark the Relationship between Sugar-Sweetened Beverage Intake and Blood Pressure in Adolescent Girls: A Metabolomics Analysis in the ELEMENT Cohort

**DOI:** 10.3390/metabo9050100

**Published:** 2019-05-17

**Authors:** Wei Perng, Lu Tang, Peter X. K. Song, Michael Goran, Martha Maria Tellez Rojo, Alejandra Cantoral, Karen E. Peterson

**Affiliations:** 1 Department of Epidemiology, Colorado School of Public Health, Anschutz Medical Center, Aurora, CO 80045, USA; 2 Lifecourse Epidemiology of Adiposity and Diabetes Center, Anschutz Medical Center, Aurora, CO 80045, USA; 3 Department of Biostatistics, University of Pittsburgh School of Public Health, Pittsburgh, PA 15261, USA; lutang@pitt.edu; 4 Department of Biostatistics, University of Michigan School of Public Health, Ann Arbor, MI 48105, USA; pxsong@umich.edu; 5 Program for Diabetes and Obesity, The Saban Research Institute, Children’s Hospital of Los Angeles, Los Angeles, CA 90027, USA; goran@usc.edu; 6 Center for Research on Nutrition and Health, National Institute of Public Health, Cuernavaca 62100, México; mmtellez@insp.mx (M.M.T.R.); acantoral@hotmail.com (A.C.); 7 CONACYT, National Institute of Public Health, Center for Research on Nutrition and Health, Cuernavaca, Morelos 62100, Mexico; 8 Department of Nutritional Sciences, University of Michigan School of Public Health, Ann Arbor, MI 48105, USA; karenep@umich.edu

**Keywords:** sugar-sweetened beverages, adolescents, metabolomics, LASSO, metabolic risk, blood pressure, uric acid, urate, nonanoate

## Abstract

We sought to identify metabolites that mark the relationship of sugar-sweetened beverage (SSB) intake with adiposity and metabolic risk among boys (*n* = 114) and girls (*n* = 128) aged 8–14 years. We conducted the analysis in three steps: (1) linear regression to examine associations of SSB intake (quartiles) with adiposity, glycemia, lipids, and blood pressure (BP); (2) least absolute shrinkage and selection operator (LASSO) regression to identify SSB-associated metabolites from an untargeted dataset of 938 metabolites; and (3) linear regression to determine whether SSB-related metabolites are also associated with adiposity and metabolic risk. In girls, SSB intake was associated with marginally higher BP (Q2 vs, Q1: 1.11 [−3.90, 6.13], Q3 vs. Q1: 1.16 [−3.81, 6.13], Q4 vs. Q1: 4.65 [−0.22, 9.53] mmHg systolic blood pressure (SBP); *P*-trend = 0.07). In boys, SSB intake corresponded with higher C-peptide insulin resistance (Q2 vs. Q1: 0.06 [−0.06, 0.19], Q3 vs. Q1: 0.01 [−0.12, 0.14], Q4 vs. Q1: 0.17 [0.04, 0.30] ng/mL; *P*-trend = 0.03) and leptin (*P*-trend = 0.02). LASSO identified 6 annotated metabolites in girls (5-methyl-tetrohydrofolate, phenylephrine, urate, nonanoate, deoxyuridine, sn-glycero-3-phosphocholine) and 3 annotated metabolites in boys (2-piperidinone, octanoylcarnitine, catechol) associated with SSB intake. Among girls, urate and nonanoate marked the relationship of SSB intake with BP. None of the SSB-associated metabolites were related to health outcomes in boys.

## 1. Introduction

Self-reported dietary assessment methods widely-used in epidemiological studies, such as food frequency questionnaires, 24-h dietary recalls, and food records, are subject to recall errors. In some instances, such as the famous example of obesity-related under-reporting of usual dietary intake [1], the recall errors are differential with respect to biological conditions and health outcomes, thereby making it difficult to account for such error in the statistical analysis. Dietary biomarkers provide a more objective assessment of dietary intake [2]. For example, urinary sodium and nitrogen have been used as biomarkers of salt and protein intake, and fatty acid composition of subcutaneous adipose tissue provides an estimate of long-term polyunsaturated fatty acid intake [3]. To date, most studies have assessed biomarkers of single foods or nutrients, rather than food groups, which may provide a more accurate reflection of how we consume foods in real life.

One food group of particular interest in the realm of obesity research is sugar-sweetened beverages (SSBs), which typically comprises non-diet sodas, sweetened fruit juices, and other beverages with added sugars (i.e., tea with sugar, coffee with sugar). Accurately assessing SSB intake is challenging given that under-reporting of intake is a particular problem for socially undesirable foods and beverages [4], an issue that may be further compounded by obesity-related underreporting of dietary intake. To address this challenge, a few studies have attempted to identify biomarkers of sugar intake, such as urinary sugar excretion, and δ13C in finger-stick blood, serum, red blood cells and hair. However, these biomarkers exhibited only modest associations with sugar intake [5].

The recent advent of high-throughput technologies has made it possible for researchers to identify novel biomarkers of dietary intake via comprehensive metabolomics profiling of tissues and fluids. Using a discovery and validation design, Gibbons et al. [6] used data from 565 participants of the Irish National Adult Nutrition Survey to identify urinary metabolites (quantified via targeted nuclear magnetic resonance [NMR] spectroscopy) associated with SSB intake, then validated the findings among 10 adults attending University College Dublin. The investigators identified four compounds of interest (formate, citrulline, taurine, and isocitrate), all of which were on carbohydrate metabolism pathways. While we are not aware of metabolomics analyses of SSB intake in youth, Mayengbam et al. [7] examined postprandial serum metabolomic correlates of B-vitamin fortified beverage consumption among 20 adolescents. The authors noted perturbations in serum homocysteine, betaine, vitamins B6 and B12, choline, folate and taurine [7]. Together, these studies support the possibility of using metabolomics to identify biomarkers of beverage intake, and provide proof-of-principle evidence of specific metabolic effects of beverage composition on circulating metabolites in youth. Yet, a missing piece revolves around understanding whether metabolites of interest (i.e., those related to SSB intake) are also associated with known risk factors for obesity-related conditions.

In this study, we leveraged data from untargeted metabolomics profiling of fasting blood, a systemic tissue that reflects all ongoing physiological processes, to identify novel metabolite biomarkers of sugar-sweetened beverage (SSB) intake that may also mark the relationship between SSBs and a range of adverse metabolic health outcomes among adolescents in Mexico City, MX.

## 2. Results

Median age of the participants was 10.0 years (range: 8.1, 14.7) and a little less than half of the sample (47.1%; *n* = 114) were boys. The majority of children reported having consumed SSBs at least four servings of sugar-sweetened beverages (SSB) in a day (63.9% of girls and 59.7% of boys; Table 1). In concordance with a previous publication in this cohort [8], the main contributors to the SSB food group were soda and fruit juices with added sugar (data not shown, available upon request). Table S1 in supplementary materials shows mean ± standard deviation (SD) of the adiposity indicators and metabolic biomarkers for boys and girls, separately.

Table 2 shows associations of quartiles of SSB intake with biomarkers of glycemia, lipid profile, adiposity, and blood pressure for boys and girls, separately. In boys, higher SSB intake was associated with higher C-peptide (Q4 vs. Q1 of SSB intake: 0.72 [95% confidence interval (CI): 0.16, 1.27] ng/mL; *P*-trend across quartiles = 0.04), C peptide insulin resistance (CP-IR) (Q4 vs. Q1 of SSB intake: 0.17 [95% CI: 0.04, 0.30] ng/mL; *P*-trend across quartiles = 0.03), and leptin (Q4 vs. Q1 of SSB intake: 3.78 [95% CI: 0.49, 7.08] ng/mL; *P*-trend across quartiles = 0.02). We also noted marginally significant positive associations with measures of fat distribution (waist circumference and sum of the subscapular and triceps skinfolds (SS+TR)), and positive associations with blood pressure. Among girls, the only association we detected was a marginal positive association with blood pressure (Table 2).

In Table 3, we show results of least absolute shrinkage and selection operator (LASSO) regression, which identified the strongest metabolite correlates of SSB intake, along with feature characteristics and annotation methods for the compounds of interest. The procedure identified 18 metabolites in girls that met the selection cut-off of 70 (i.e., in the 100 bootstrap replication procedures, these metabolites were selected at least 70% of the time in the LASSO models), six of which were named compounds: 5-methyl-tetrohydrofolate (5-THF), phenylephrine, urate, nonanoate, deoxyuridine, and sn-glycero-3-phosphocholine. In boys, 11 compounds met the selection cut-off, three of which were named compounds: 2-piperidinone, octanoylcarnitine, and catechol. 

In Table 4, we show associations of the named metabolites from Table 3 with the adiposity indicators and metabolic biomarkers. In girls, urate and nonanoate were each positively associated with blood pressure. A 1 z-score increment in urate was associated with 3.09 (95% CI: 1.38, 4.79) mmHg higher systolic blood pressure (SBP), and 1.31 (95% CI: 0.01, 2.61) mmHg higher diastolic blood pressure (DBP). Similarly, a 1 z-score increment in nonanoate was associated with 2.27 (95% CI: 0.58, 3.97) mmHg higher SBP. None of the named metabolites from Table 3 were associated with outcomes of interest in boys (Table 4).

Based on our findings of the relevance of uric acid as a potential marker for the relationship between SSB intake and hypertension in girls, we further adjusted all conventional models (i.e., excluding LASSO) for total energy-adjusted fish and organ meat intake, as both of these foods are purine sources that contribute to hyperuricemia. Inclusion of these food groups did not change our results. For example, in girls, using the same covariates as in models for Table 2, but with inclusion of fish intake as a covariate, the estimate for the Q4 vs. Q1 of SSB intake in relation to SBP was 5.17 (95% 0.45, 9.89) mmHg (*P*-trend across quartiles = 0.04). Likewise, in the model relating urate as the independent variable to SBP as the outcome in girls, the estimate for urate was 2.90 (95% CI: 1.19, 4.60) mmHg. 

## 3. Discussion

In this study of 242 Mexican youth, we sought to identify metabolites that mark the relationship of sugar-sweetened beverage (SSB) intake with adiposity and metabolic risk. SSB intake was associated with higher blood pressure in girls, and with biomarkers of glycemia (C-peptide, CP-IR, leptin) and fat distribution (waist circumference, skinfold thicknesses) in boys. In girls, urate (aka uric acid) and nonanoate (aka nonanoic acid) marked the relationship between SSB intake and blood pressure. We did not identify any metabolites that marked associations of SSB intake with the adiposity or metabolic risk biomarkers in boys.

### 3.1. Girls

In girls, higher SSB intake was associated with higher systolic and diastolic blood pressure. We identified urate and nonanoate as key metabolites that mark this relationship. Our finding with respect to urate corroborates a longstanding literature on the relationship between sugar (in particular, fructose) intake and gout [9], a condition caused by high serum uric acid in tissues (hyperuricemia), as well as the established link between hyperuricemia and high blood pressure in adults [10] and children [11,12]. The process of phosphorylation of fructose, the first step of fructose metabolism, leads to an increase in circulating uric acid as a byproduct [13]. Uric acid is an antioxidant that initially exerts neuroprotective functions. However, when present in excess, this compound has detrimental effects on health via induction of platelet aggregation and chronic systemic inflammation [14], both of which are precursors to elevated blood pressure [15,16]. Recent meta-analyses showed a significant association of serum uric acid and incident hypertension, independent of traditional risk factors (i.e., smoking habits, age, lipid profile, physical activity levels, and socioeconomic characteristics) [17,18]. The fact that we identified an association only in females may be related to the effects of SSB on female reproductive hormones [19], which have a physiological impact on circulating uric acid [20]. In addition to the above explanations, we acknowledge the possibility that the association of SSB intake with uric acid in girls may be confounded or modified by the effect of menstrual cycle hormones.

Nonanoate also marked the SSB/blood pressure association in girls. Nonanoate is a flavoring agent used predominantly in alcoholic drinks but may also be added to fruit-flavored beverages, such as the sweetened fruit juices inquired about by our food frequency questionnaire (FFQ) instrument. While we were not able to locate any published studies on associations of nonanoate with blood pressure, methyl nonanoate (a compound that is not yet annotated in the chemical library of the laboratory that performed the metabolomics analysis) interacts with neutrophil gelatinase-associated lipocalin (NGAL), an iron-trafficking protein involved in renal development [21]. Given that the kidneys play a key role in pressor systems, nonanoate-NGAL interactions is one possible pathway through which nonanoate may influence blood pressure.

We also identified several metabolites associated with SSB intake that were not associated with blood pressure, but are worth mentioning because they shed light biochemical pathways that may link SSB intake to adverse health outcomes not yet detectable in adolescents. SSB intake was inversely related to 5-methyl-THF, a relationship that may transpire from interactions between glucose metabolism pathways and the folic acid cycle [22]. We also noted a positive association of SSB intake with: phenylephrine, an exogenous adrenergic receptor agonist that causes vasoconstriction; deoxyuridine, a nucleoside involved in DNA synthesis; and sn-glycero-3-phosphocholine, a phospholipid intermediate in the catabolism of lecithin—an emulsifier and stabilizer used in beverages.

### 3.2. Boys

In boys, higher SSB intake was associated with higher fasting C-peptide, CP-IR, and leptin; higher central adiposity (waist circumference); and higher blood pressure. While these relationships align with an established literature on the detrimental effects of SSB consumption on obesity and cardiometabolic risk [23], none of the three named SSB-related metabolites were associated with the aforementioned health outcomes. The null findings in this regard may be due to our stringent criteria for retention of metabolites in the LASSO regression. 

Despite these null results, SSB intake was associated with three named metabolites in boys that may provide insights into the physiological consequences of SSB consumption. SSB intake was positively associated with 2-piperidinone, an organic compound isolated from black pepper used as a reagent in synthesis of other organic compounds, including pharmaceuticals [24]; and catechol, an inorganic compound found in cocoa powder and beverages [25]. On the other hand, SSB intake was negatively associated with octanoylcarnitine, a medium-chain acylcarnitine that has been implicated in metabolic conditions with altered fatty acid β-oxidation [26,27]. Some of these metabolites may be related to SSBs directly through intake (i.e., 2-piperidone via coffee and hot cocoa consumption), or via physiological effects of SSB consumption (i.e., glucose and fructose intake can interfere with fatty acid oxidation [28], resulting in disturbances to intermediates of lipid metabolism like octanoylcarnitine).

### 3.3. Strengths and Weaknesses

This study has several strengths. First, we used bootstrap LASSO, a multivariate dimension reduction technique that reduces the possibility of false positive findings, an ongoing challenge of ’omics analyses. Second, we used research-quality measures of body composition and metabolic biomarkers to assess outcomes of interest in this study. Third, in the Early Life Exposure in Mexico to Environmental Toxicants (ELEMENT) project, we have rich data on key covariates including physician-assessed pubertal status and other lifestyle and dietary characteristics to control for confounding and assess for effect modification.

However, our study is not without limitations. First, our sample size was relatively small, which may have reduced our ability to detect associations, although we note that our N is comparable to that of other untargeted metabolomics analyses carried out in children and adolescents [29,30]. Second, the SSB beverage group comprises beverages that contain more than just their sweeteners. Thus, we cannot make firm conclusions regarding specific ingredients responsible for the SSB-metabolite associations we detected. Third, use of a predictive algorithm like LASSO to identify metabolite predictors of SSB intake does not reflect the hypothesized direction biological relationship (i.e., SSB intake affects circulating metabolites). Thus, the procedure may not have appropriately accounted for the covariance structure among the SSB-metabolite associations. However, LASSO assesses conditional associations of each metabolite with the health outcomes (i.e., the relationship of each metabolite with an outcome while adjusting for all other metabolites in the dataset), which to some extent, handles intercorrelations among metabolites. Fourth, given that there are currently no standards on the optimal selection factor for bootstrap LASSO, the one we selected (≥70) was arbitrary. Fifth, we cannot be certain that SSBs are the definitive source of metabolites of interest. For example, many over-the-counter cold medications and decongestants contain phenylephrine. Finally, the cross-sectional design impedes our ability to ascertain temporality. Finally, assessment of the metabolites took place at a single time-point and thus, we are not able to make inference on metabolic homeostasis. 

## 4. Materials and Methods

### 4.1. Study Population

This study included children and adolescents in the ELEMENT project, a cohort of mother-child dyads in Mexico City, Mexico [31]. The present analysis draws from a subset of the children (*n =* 250) who were recruited in 2010 for a follow-up study if they were 8–14 years of age, and had adequate volumes of archived prenatal biospecimens for laboratory assays. At research visits that took place in 2010, the children provided an 8-h fasting blood sample and participated in anthropometric assessment. Our study sample included 242 children with dietary intake data on SSBs, anthropometry or metabolic biomarkers, and adequate fasting serum volume for metabolomics analyses. The institutional review boards of the Mexico National Institute of Public Health and the University of Michigan approved the research protocols (IRB #HUM00034344). 

### 4.2. Dietary Assessment

At the 8- to 14-year research visit, research staff administered an age-specific semi-quantitative food frequency questionnaire (FFQ) to the children. The FFQ was adapted from the 2006 Mexican Health and Nutrition Survey [32], and queried frequency of consumption of 109 food items during the last seven days. Participants reported their frequency of consumption of standard portions of each food or beverage ranging from “Never” to “≥6 times per day.”

Prior to formal data analyses, we combined the 109 food items into 35 food groups (details previously published) [33], including the SSB group which comprised non-diet sodas, fruit juices with added sugar (did not include natural fruit juices), other beverages with added sugar namely, coffee, tea, and water. The components of this food group were selected based on the predominant use of fructose and/or glucose as sweeteners, and previous findings in this cohort of a specific detrimental effect of consumption of this specific SSB food group on obesity-related health outcomes during childhood and adolescence [8] and young adulthood [34]. For each food group, we estimated total daily energy intake of each food group using the United States Department of Agriculture Food Composition Database [35] and adjusted each food group by total energy intake using the residual method [36]. 

### 4.3. Untargeted Metabolomics Profiling

The Michigan Regional Comprehensive Metabolomics Resource Core (MRC^2^) carried out untargeted metabolomic profiling in fasting serum collected from the participants at the 8- to 14-year research visit. MRC^2^’s untargeted platform utilizes liquid chromatography and mass spectrometry (LC/MS). Details on the laboratory procedures are included in Appendix A. The procedure yielded 9303 chemical features. After removal of redundant compounds and those with >70% of values missing, the final data set comprised 938 unique compounds, 332 of which were annotated (named) metabolites whose spectral peaks, fragmentation patterns, and retention times matched with compounds within the laboratory’s chemical library. Upon receiving the data from the lab, we removed run-order batch effects by adjusting batch-specific median deviances for the global median and used a non-parametric LOESS smoothing curve to account for deviation over time, imputed values below the detection using the *K*-nearest neighbor algorithm (*K* = 5) using the *IMPUTE* package in R, and standardized each metabolite as a z-score using a rank-based inverse normal transformation.

Of note, in the multivariate dimension-reduction analysis, we included all 938 metabolites, both named and un-named. Our rationale for taking this approach is that un-named compounds represent reliably detected metabolites that contribute to the correlation structure among metabolites in the dataset, and thus, including these metabolites in the analysis more closely reflects true biological associations. However, our interpretation of results focuses on the annotated compounds, as we have previously [37]. 

### 4.4. Adiposity and Conventional Biomarkers of Metabolic Risk

#### 4.4.1. Adiposity

We measured the children’s weight (kg) on a digital scale (BAME Mod 420; Catálogo Médico), height (cm) using a calibrated stadiometer (BAME Mod 420; Catálogo Médico), waist circumference (cm) using a non-stretchable measuring tape (QM2000; QuickMedical), and the subscapular (SS) and triceps (TR) skinfold thicknesses (mm) using calibrated skin calipers (Lange; Beta Technology) [38]. 

We used the weight and height measurements to calculate body mass index (BMI) [39], then used the World Health Organization growth reference to calculate BMI z-score [40]. We used waist circumference as a proxy for central visceral adiposity [39], and the sum (SS+TR) of the subscapular and triceps skinfolds as a marker of subcutaneous adiposity [41].

#### 4.4.2. Blood Pressure

Research staff used an automated blood pressure monitor (BpTRU; Coquitlam, BC) to measure systolic (SBP) and diastolic blood pressure (DBP) five times. The intra-class correlation (ICC) between the measurements were high (ICC_SBP_ = 0.95; ICC_DBP_ = 0.89), so we took the average of the values in the statistical analysis.

#### 4.4.3. Glycemia Biomarkers

We assayed all glycemia biomarkers in fasting serum. Glucose was measured enzymatically, and C-peptide was assayed using an automated chemiluminescence immunoassay (Immulite 1000, Siemens Medical Solutions). These biomarkers serve as assessments of glycemic control: fasting glucose is an indicator of glucose metabolism, and C-peptide is a proxy for insulin secretion [42]. We measured leptin from serum using a radioimmunoassay (Millipore).

#### 4.4.4. Lipid Profile

We measured total cholesterol, triglycerides, and high-density lipoprotein cholesterol (HDL-C) in peripubertal fasting serum samples (mg/dL) using a biochemical analyzer (Cobas Mira Plus, Roche Diagnostics), and calculated low-density lipoprotein cholesterol (LDL-C) as: Total cholesterol − HDL-C − (Triglycerides/5). 

### 4.5. Covariates

At enrollment, mothers reported on age, reproductive history, lifestyle and sociodemographic characteristics. At the peripubertal visit, a trained pediatrician assessed each child to determine Tanner stage on a scale of 1 (no development) to 5 (full development) for genital (boys), breast (girls), and pubic hair (both) development [43]. We then dichotomized pubertal status as pre-pubertal vs. pubertal: boys were classified as pubertal if they received an assessment of Tanner stage >1 for genital or pubic hair development, and girls were classified as pubertal if they received an assessment of Tanner stage >1 for breast or pubic hair development [44]. At this visit, the child (with proxy-assistance from the accompanying caregiver when necessary) completed a validated interviewer-administered questionnaire that queried the amount of time he/she spent engaged in moderate-to-vigorous physical activities each week [45]. For the analysis, we parameterized physical activity as quartiles of total hours per week as we have in previous studies [33].

### 4.6. Data Analysis

Prior to formal analysis, we examined bivariate associations of total energy-adjusted SSB intake as a continuous variable across background characteristics of the study sample. This step, in conjunction with our a priori knowledge of determinants of metabolic risk in youth, informed our selection of covariates for multivariable models. Due to known sex-specific differences in metabolism in the age range of our study sample [44], as well as sex-specific effects of dietary intake on health outcomes in this population [33], we implemented all models separately for boys and girls.

We carried out the main analysis in three steps. First, we examined associations of quartiles of SSB intake (independent variable) with the adiposity indicators and conventional metabolic biomarkers (dependent variables) to ascertain relationships of SSB intake with the health outcomes. Next, we identified metabolite biomarkers of SSB intake using LASSO regression [46]. Finally, we examined whether the metabolites of interest are also associated with health outcomes “predicted” by SSB intake in the first step. We describe each step in detail, below.

In the first step of our analysis, we examined sex-specific associations of quartiles of SSB intake with the conventional biomarkers of glycemia, adiposity, and blood pressure using linear regression models that accounted for the child’s age and pubertal status. In the models, we interpreted the estimates for the 2nd, 3rd, and 4th quartiles of intake vs. the 1st quartile as the reference, and also tested for a linear trend by entering an indicator for quartiles of SSB intake as a continuous variable. In this analysis, we considered a relationship to be of interest for further exploration in downstream analysis if the *P*-trend across the SSB quartiles <0.10. 

In the second step, we identified metabolite biomarkers of SSB intake using LASSO regression [46]. In the LASSO model, we included all 938 metabolites in the model as predictors, and entered quartiles of SSB intake as the outcome. In the context of association analysis (as is the case here), the direction of the predictor/outcome relationship is exchangeable such that statistical methods to unveil associations are invariant to conceptual relationships. Thus, we capitalized on this proven dimension-reduction technique to identify the strongest SSB/metabolite associations. LASSO is a regularized regression technique that detects the strongest predictor-outcome signals from a high-dimensional and correlated set of predictors [46]. The key feature of LASSO is the imposition of a tuning parameter on model coefficients (*β*, representing the relationship between a given set of metabolite and SSB intake) while conditioning on all other metabolites and adjusting for the covariates (age and pubertal status). The tuning parameter shrinks weak or null *β*s to zero and effectively removes them from the predictive model [47]. We used 10-fold cross validation to identify the threshold below which a beta estimate is shrunk to 0 (based on lowest model validation error). We then conducted bootstrap LASSO, which entailed creation of 100 copies of the original metabolite data set, and applying the LASSO procedure to each resampled dataset. We then identified metabolites of interest as those that were selected by LASSO at least 70% of the time (i.e., selection factor ≥70) [48]. We used this arbitrary threshold based on a relaxation of the more stringent one used by Bach et al. (selection factor ≥70) [48] due to lower model error, and to capture a larger number of annotated compounds in the final predictive model for interpretability purposes.

In the third step, we investigated whether the SSB-associated metabolites are also associated with the adiposity indicators and metabolic biomarkers that were associated with SSB intake in the first step. Here, we used multivariable linear regression models that accounted for age and pubertal status. We considered a metabolite to be associated with a conventional biomarker of interest if the *P*-value for the β coefficient for the relationship between the metabolite and the biomarker was <0.10.

We also carried out sensitivity analysis where we further adjusted multivariable models (i.e., those in Steps 1 and 3) for physical activity level, parity, and maternal smoking during pregnancy, as each of these may be associated with dietary intake as well as obesity-related health outcomes. In addition, based on our results, we also further explored the impact of adjustment for total energy adjusted fish and organ meat intake, as each of these foods were on related biochemical pathways identified in the main analysis. Inclusion of these variables did not materially change our results, thus we did not include them in the final models for the sake of parsimony and statistical power.

Unless otherwise stated, we performed statistical analyses using SAS 9.4 (Cary, NC, USA).

## 5. Conclusions

In this study of Mexican children and adolescents, we identified urate (uric acid) and nonanoate as metabolites associated with SSB intake that were also associated with higher blood pressure in girls. These findings may have ramifications for understanding the pathophysiology of obesity-related conditions that manifest with a cluster of cardiometabolic disturbances that are of particular concern in Hispanic populations (i.e., non-alcoholic fatty acid disease) due to evidence of synergistic gene-diet interactions [49]. Future prospective studies are necessary to establish temporal relations of SSB intake, metabolites, and conventional metabolic risk biomarkers in this cohort, as well as other populations.

## Figures and Tables

**Table 1:** Percent (% [N]) of participants reporting intake of any sugar-sweetened beverages among 242 Early Life Exposure in Mexico to Environmental Toxicants (ELEMENT) project participants.

Intake of Any Sugar-Sweetened Beverage ^a^	Girls *n* = 128	Boys *n* = 114
Never to <1 time/month	0.0% (0)	4.4% (5)
1–3 times/month	4.7% (6)	2.6% (3)
Once per week	8.6% (11)	4.4% (5)
2–4 times/week	3.9% (5)	2.6% (3)
5–6 times/week	3.9% (5)	12.3% (14)
Once per day	4.7% (6)	6.1% (7)
2–3 times/day	10.9% (14)	7.9% (9)
>4 times/day	63.3% (81)	59.7% (68)

^a^ Defined as non-diet soda, coffee with sugar, tea with sugar, sweetened fruit juice (does not include natural fruit juices), or any drink with added sugar.

**Table 2:** Associations of quartiles of sugar-sweetened beverage (SSB) intake with metabolic biomarkers in ELEMENT participants during peripuberty.

-	Associations (β ^a^ [95% Confidence Interval (CI)]) of Quartiles of SSB Intake with Adiposity and Metabolic Risk Biomarkers during Peripuberty			
	Girls (*n* = 128)			
	Q2 vs. Q1	Q3 vs. Q1	Q4 vs. Q1	*P*-Trend
	*n* = 32 vs. 32	*n* = 32 vs. 32	*n* = 32 vs. 32	
Glycemia				
Fasting glucose (mg/dL)	0.02 (−5.09, 5.12)	0.91 (−4.19, 6.00)	−0.13 (−5.23, 4.98)	0.95
Fasting C-peptide (ng/mL)	0.12 (−0.49, 0.74)	−0.23 (−0.85, 0.38)	0.04 (−0.57, 0.65)	0.81
CP-IR ^b^	0.05 (−0.14, 0.24)	−0.04 (−0.23, 0.14)	0.04 (−0.15, 0.23)	0.94
Leptin (ng/mL)	−1.33 (−6.04, 3.39)	−3.37 (−8.07, 1.34)	−0.72 (−5.43, 4.00)	0.58
Lipid Profile				
Total cholesterol (mg/dL)	0.85 (−11.77, 13.47)	6.29 (−6.31, 18.88)	0.14 (−12.48, 12.76)	0.77
HDL (mg/dL)	2.81 (−2.83, 8.45)	2.42 (−3.22, 8.05)	−1.14 (−6.78, 4.50)	0.68
LDL (mg/dL)	−3.89 (−13.99, 6.20)	3.14 (−6.93, 13.22)	−2.57 (−12.66, 7.53)	0.98
Triglycerides (mg/dL)	9.67 (−13.30, 32.63)	3.61 (−19.31, 26.53)	19.24 (−3.72, 42.19)	0.17
Adiposity and Blood Pressure				
BMI z-score ^c^	0.11 (−0.48, 0.70)	−0.20 (−0.79, 0.39)	0.20 (−0.39, 0.79)	0.76
Waist circumference (cm)	0.30 (−4.65, 5.25)	−1.33 (−6.27, 3.61)	2.04 (−2.90, 6.99)	0.58
SS+TR (mm)	−0.37 (−5.86, 5.12)	−1.64 (−7.12, 3.84)	1.50 (−3.99, 6.99)	0.72
SBP (mmHg)	1.11 (−3.90, 6.13)	1.16 (−3.81, 6.13)	4.65 (−0.22, 9.53)	0.07
DBP (mmHg)	0.02 (−3.61, 3.66)	1.27 (−2.33, 4.86)	3.08 (−0.45, 6.62)	0.07
-	**Boys (*n* = 114)**			
	**Q2 vs. Q1**	**Q3 vs. Q1**	**Q4 vs. Q1**	***P*-Trend**
	***n* = 29 vs. 29**	***n* = 28 vs. 29**	***n* = 28 vs. 29**	
Glycemia				
Fasting glucose (mg/dL)	1.12 (−2.80, 5.03)	1.70 (−2.27, 5.67)	1.52 (−2.43, 5.47)	0.42
Fasting C-peptide (ng/mL)	0.27 (−0.28, 0.83)	0.00 (−0.57, 0.56)	0.72 (0.16, 1.27)	0.04
CP-IR ^b^	0.06 (−0.06, 0.19)	0.01 (−0.12, 0.14)	0.17 (0.04, 0.30)	0.03
Leptin (ng/mL)	0.99 (−2.27, 4.25)	1.78 (−1.53, 5.09)	3.78 (0.49, 7.08)	0.02
Lipid profile				
Total cholesterol (mg/dL)	−1.20 (−15.68, 13.28)	3.37 (−11.32, 18.07)	−2.39 (−17.01, 12.22)	0.90
HDL (mg/dL)	−0.49 (−6.61, 5.62)	−1.24 (−7.44, 4.96)	−3.83 (−10.00, 2.34)	0.22
LDL (mg/dL)	−2.70 (−14.80, 9.41)	2.90 (−9.39, 15.18)	0.26 (−11.96, 12.48)	0.76
Triglycerides (mg/dL)	9.97 (−9.76, 29.70)	8.58 (−11.44, 28.61)	5.89 (−14.02, 25.81)	0.61
Adiposity and blood pressure				
BMI z-score ^c^	0.40 (−0.21, 1.00)	0.13 (−0.48, 0.74)	0.55 (−0.05, 1.16)	0.15
Waist circumference (cm)	3.81 (−0.86, 8.48)	1.69 (−3.05, 6.42)	5.06 (0.35, 9.77)	0.08
SS+TR (mm)	2.69 (−2.80, 8.18)	0.83 (−4.74, 6.40)	5.51 (−0.03, 11.06)	0.10
SBP (mmHg)	4.18 (−0.90, 9.26)	7.08 (2.00, 12.16)	8.79 (3.69, 13.90)	0.0004
DBP (mmHg)	3.88 (0.24, 7.51)	6.18 (2.55, 9.81)	7.10 (3.45, 10.75)	<0.0001

^a^ Estimates are adjusted for child’s age and pubertal status (breast and pubic hair for girls; testicular volume and pubic hair for boys); ^b^ Calculated as [fasting serum C-peptide × fasting serum glucose]/405; ^c^ According to the WHO sex-specific growth reference for children 5–19 years of age.

**Table 3:** Metabolites associated with quartiles of sugar-sweetened beverage (SSB) intake, selected via least absolute shrinkage and selection operator (LASSO) regression.

-	Feature Characteristics and Annotation Methods					LASSO Parameters	
	Ion Acquisition Mode	Retention Time (min)	*m/z*	Annotation Method	Level of Confidence	Selection	β ^a^
Girls (*n* = 128)							
5-methyl-tetrohydrofolate (THF)	+	3.63	460.194	In-house library	Level 1	96	−0.508
Urate	+	1.00	169.037	In-house library	Level 1	96	0.576
Unknown	+	9.54	920.466			95	−0.467
Phenylephrine	+	0.94	168.102	In-house library	Level 1	95	0.419
Unknown	+	29.63	647.560			94	−0.346
Unknown	−	0.79	195.810			93	0.399
Unknown	+	24.97	810.597			90	−0.348
Unknown	+	10.11	211.131			83	0.249
Unknown	+	22.22	435.271			81	−0.288
Nonanoate	−	18.33	157.123	In-house library	Level 1	81	0.287
Unknown	−	18.57	473.275			80	0.335
Unknown	−	8.47	516.006			78	−0.247
Unknown	+	22.91	359.317			77	−0.238
Unknown	+	19.33	501.317			76	−0.241
Unknown	−	16.19	398.036			75	0.265
Deoxyuridine	+	1.67	251.066	In-house library	Level 1	75	0.257
Unknown	+	19.80	588.318			73	−0.269
Unknown	−	20.63	584.236			73	0.198
Unknown	+	25.07	1614.146			72	−0.216
Sn-glycero-3-phosphocholine	+	0.61	258.111	In-house library		71	0.192
Boys (*n* = 114)							
2-piperidinone	+	2.68	100.077	In-house library	Level 1	96	0.45
Unknown	−	12.73	430.015			91	0.36
Unknown	+	18.77	455.202			91	0.45
Unknown	+	7.82	279.171			90	0.51
Octanoylcarnitine	+	25.31	752.562	In-house library	Level 1	86	−0.42
Unknown	+	11.65	311.147			86	−0.38
Catechol	−	2.50	109.029	In-house library	Level 1	82	0.35
Unknown	−	24.00	883.537			81	0.30
Unknown	+	14.05	793.411			77	−0.28
Unknown	+	22.91	471.353			75	0.24
Unknown	+	12.03	1238.497			75	−0.37

^a^ Estimates are adjusted for age and pubertal status (breast and pubic hair for girls; testicular volume and pubic hair for boys).

**Table 4:** Associations of quartiles of SSB-related metabolites with blood pressure in ELEMENT participants during peripuberty.

-	β ^a^ (95% CI) per 1-SD Increment in Each SSB-Related Metabolite with Select Adiposity Indicators and Metabolic Biomarkers					
	Girls (*n* = 128)					
	5-Methyl-THF	Phenylephrine	Urate	Nonanoate	Deoxyuridine	Sn-Glycero-3-Phosphoholine
Blood pressure						
SBP (mmHg)	−1.18 (−2.92, 0.55)	0.03 (−1.68, 1.73)	3.09 (1.38, 4.79)	2.27 (0.58, 3.97)	0.04 (−1.73, 1.81)	−0.10 (−1.77, 1.57)
DBP (mmHg)	−0.44 (−1.73, 0.84)	0.26 (−1.00, 1.52)	1.31 (0.01, 2.61)	0.31 (−0.98, 1.60)	0.44 (−0.86, 1.75)	−0.05 (−1.28, 1.19)
-	**Boys (*n* = 114)**					
	**2-Piperidinone**		**Octanoylcarnitine**		**Catechol**	
Metabolic biomarkers						
Fasting C-peptide (ng/mL)	0.06 (−0.13, 0.25)		−0.15 (−0.37, 0.08)		0.08 (−0.15, 0.30)	
CP-IR ^b^	0.01 (−0.03, 0.06)		−0.03 (−0.08, 0.02)		0.02 (−0.03, 0.07)	
Leptin (ng/mL)	0.79 (−0.32, 1.89)		0.19 (−1.11, 0.15)		0.06 (−1.28, 1.39)	
Adiposity						
BMI z-score	0.16 (−0.05, 0.36)		−0.04 (−0.28, 0.20)		0.01 (−0.23, 0.25)	
Waist circumference (cm)	1.50 (−0.07, 3.07)		−0.37 (−2.24, 1.50)		0.26 (−1.64, 2.16)	
SS+TR (mm)	1.35 (−0.50, 3.21)		−0.62 (−2.81, 1.57)		−0.27 (−2.50, 1.96)	
Blood pressure						
SBP (mmHg)	0.98 (−0.86, 2.83)		0.37 (−1.68, 2.43)		1.98 (−0.06, 4.02)	
DBP (mmHg)	1.10 (−0.23, 2.43)		−0.98 (2.46, 0.51)		1.23 (−0.26, 2.72)	

^a^ Estimates are adjusted for child’s age and pubertal status based on breast and pubic hair development for girls, and based on testicular volume and public hair development in boys. ^b^ Calculated as [fasting serum C-peptide × fasting serum glucose]/405.

## Supplementary Materials

The following are available online at https://www.mdpi.com/2218-1989/9/5/100/s1, Table S1: Descriptive statistics of age, metabolic outcomes, and pubertal status for 242 ELEMENT participants.

## Appendix A. Liquid Chromatography–Mass Spectrometry (LC–MS) Methods

### Appendix A.1. Chemicals and Reagents

Water, methanol, acetonitrile, formic acid, ammonium bicarbonate, ammonium hydroxide solution, and ammonium acetate (99.999%) were purchased from Sigma-Aldrich (St. Louis, MO) and were liquid chromatography–mass spectrometry (LC–MS) grade except as noted.

### Appendix A.2. Sample Preparation

Plasma samples were thawed on ice prior to processing. For deproteinization in preparation for LC-MS analysis, 100 µL of plasma was combined with 400 µL 1:1:1 methanol:acetone:water containing the following internal standards (l-(D4) thymine, l-[^15^N] anthranilic acid (each 5 uM); L-(^15^N)_2_ tryptophan, gibberellic acid, l-epibrassinolide (each 20 uM) for metabolites recovery assesment. The sample was vortexed, then centrifuged (10 min at 15,000 × *g*). For reversed phase (RPLC)-MS analysis, the supernatant was transferred to a clean vial and dried under a stream of nitrogen gas. The dried sample was reconstituted 50 µL MeOH: Water (50:50) containing zeatin (1 uM) as an instrument performance standard. All samples were processed in random order and were assigned to a random LC–MS run order using a computerized algorithm.

### Appendix A.3. Optimized LC–MS Methods

For reversed-phase liquid chromatography and mass spectrometry (RPLC–MS), samples were analyzed on an Agilent 1200 LC/6530 quadrupole time of flight (qTOF) MS system (Agilent Technologies, Inc., Santa Clara, CA USA) using the Waters Acquity HSS T3 1.8 µ column (Waters Corporation, Milford, MA). Each sample was analyzed twice, once in positive and once in negative ion mode. For positive ion mode runs, mobile phase A was 100% water with 0.1% formic acid and mobile phase B was 100% methanol with 0.1% formic acid. For negative ion mode runs, the formic acid was replaced with 0.1% (m/v) ammonium bicarbonate. The gradient for both positive and negative ion modes was as follows: 0–0.5 min 1%B, 0.5–2 min 1–99%B, 2–6 min 99%B, 6–6.1 min 99–1%B, hold 1%B until 9 min. The flow rate was 0.35 mL/min and the column temperature was 40 °C. The injection volume for positive and negative mode was 5 µL and 8 µL, respectively. Mass spectrometry was performed by electrospray ionization with an Agilent Jetstream ion source, with full-scan mass spectra acquired over the *m*/*z* range 50–1500 Da. Source parameters were: drying gas temperature 350 °C, drying gas flow rate 10 L/min, nebulizer pressure 30 psig, sheath gas temp 350 °C and flow 11 L/min, and capillary voltage 3500 V, with internal reference mass correction.

### Appendix A.4. Data Analysis Workflow

Chromatographic peaks that represent metabolites—which are henceforth termed “features”—were detected in the data using the automated “Find by Feature” algorithm in Agilent Masshunter Qualitative Analysis Software. Feature alignment between samples was performed using an in-house software package called “Binner” (methods paper currently under review), with annotation matching to 0.05 minutes and 2 or 3 mDa mass accuracy. Binner groups features into bins based on similarity of retention time and computes correlation of feature intensities across all samples, followed by hierarchical clustering to subdivide bins into smaller clusters. Additionally, the software computes a matrix of mass differences on a per-bin basis and compares these to a list of known adducts and neutral losses. The application of Binner resulted in a ~30% decrease in features from 6000 to 4200 after removal of signals likely to be adducts, fragments, dimers, and isotopes. We then examined a correlation matrix of retention times for the remaining 4200 features and identified feature clusters based on gaps in retention time of >0.05 minutes. Within each cluster, we selected the most intense peak to retain as a unique metabolite feature. This procedure resulted in the final data set comprising 938 unique metabolites.

Once we arrived at the final list of 938 unique features, we annotated the dataset by using MRC^2^’s in-house library of 800 known metabolite standards previously analyzed under identical LC–MS conditions. Our in-house library offers Level 1 confidence according to proposed reporting standards by the Metabolomics Standards Initiative (MSI) [50]. Features that were not identified via our in-house library were searched for possible matches using the online Metlin database (http://metlin.scripps.edu) and Human Metabolome Database (HMDB; http://www.hmdb.ca), both of which confer Level 3 confidence. In many cases, the database searches resulted in multiple possible matches for each feature within a 10 ppm mass error window. Metabolite matches were ranked in order of ascending mass error, and among matches with equivalent mass error, in order of ascending Metlin or HMDB identification (ID) number. At this point, remaining features (*n* = 606) which did not match any database entries were not considered for further evaluation and denoted as an unannotated feature in the analytic dataset.

## References

[1] Lissner L., Troiano R.P., Midthune D., Heitmann B.L., Kipnis V., Subar A.F., Potischman N. (2007). OPEN about obesity: Recovery biomarkers, dietary reporting errors and BMI. Int. J. Obes..

[2] Potischman N. (2003). Biologic and methodologic issues for nutritional biomarkers. J. Nutr..

[3] Bingham S.A. (2002). Biomarkers in nutritional epidemiology. Public Health Nutr..

[4] Subar A.F., Freedman L.S., Tooze J.A., Kirkpatrick S.I., Boushey C., Neuhouser M.L., Thompson F.E., Potischman N., Guenther P.M., Tarasuk V. (2015). Addressing Current Criticism Regarding the Value of Self-Report Dietary Data. J. Nutr..

[5] Davy B., Jahren H. (2016). New markers of dietary added sugar intake. Curr. Opin. Clin. Nutr. Metab. Care.

[6] Gibbons H., McNulty B.A., Nugent A.P., Walton J., Flynn A., Gibney M.J., Brennan L. (2015). A metabolomics approach to the identification of biomarkers of sugar-sweetened beverage intake. Am. J. Clin. Nutr..

[7] Mayengbam S., Virtanen H., Hittel D.S., Elliott C., Reimer R.A., Vogel H.J., Shearer J. (2019). Metabolic consequences of discretionary fortified beverage consumption containing excessive vitamin B levels in adolescents. PLoS ONE.

[8] Cantoral A., Tellez-Rojo M.M., Ettinger A.S., Hu H., Hernandez-Avila M., Peterson K. (2016). Early introduction and cumulative consumption of sugar-sweetened beverages during the pre-school period and risk of obesity at 8–14 years of age. Pediatric Obes..

[9] Jamnik J., Rehman S., Blanco Mejia S., de Souza R.J., Khan T.A., Leiter L.A., Wolever T.M., Kendall C.W., Jenkins D.J., Sievenpiper J.L. (2016). Fructose intake and risk of gout and hyperuricemia: A systematic review and meta-analysis of prospective cohort studies. BMJ Open.

[10] Osgood K., Krakoff J., Thearle M. (2013). Serum Uric Acid Predicts Both Current and Future Components of the Metabolic Syndrome. Metab. Syndr. Relat. Disord..

[11] Reis L.N., Reuter C.P., Pollo Renner J.D., Burgos L.T., Rech Franke S.I., Burgos M.S. (2018). High urate concentration is associated with elevated blood pressure in schoolchildren. J. Pediatric Endocrinol. Metab. JPEM.

[12] Viazzi F., Antolini L., Giussani M., Brambilla P., Galbiati S., Mastriani S., Stella A., Pontremoli R., Valsecchi M.G., Genovesi S. (2013). Serum Uric Acid and Blood Pressure in Children at Cardiovascular Risk. Pediatrics.

[13] Stirpe F., Della Corte E., Bonetti E., Abbondanza A., Abbati A., De Stefano F. (1970). Fructose-induced hyperuricaemia. Lancet.

[14] Sun D., Li S., Zhang X., Fernandez C., Chen W., Srinivasan S.R., Berenson G.S. (2014). Uric Acid Is Associated with Metabolic Syndrome in Children and Adults in a Community: The Bogalusa Heart Study. PLoS ONE.

[15] De Miguel C., Rudemiller N.P., Abais J.M., Mattson D.L. (2015). Inflammation and hypertension: New understandings and potential therapeutic targets. Curr. Hypertens. Rep..

[16] Gkaliagkousi E., Passacquale G., Douma S., Zamboulis C., Ferro A. (2010). Platelet activation in essential hypertension: Implications for antiplatelet treatment. Am. J. Hypertens..

[17] Grayson P.C., Kim S.Y., LaValley M., Choi H.K. (2011). Hyperuricemia and incident hypertension: A systematic review and meta-analysis. Arthritis Care Res..

[18] Wang J., Qin T., Chen J., Li Y., Wang L., Huang H., Li J. (2014). Hyperuricemia and risk of incident hypertension: A systematic review and meta-analysis of observational studies. PLoS ONE.

[19] Schliep K.C., Schisterman E.F., Mumford S.L., Pollack A.Z., Perkins N.J., Ye A., Zhang C.J., Stanford J.B., Porucznik C.A., Hammoud A.O. (2013). Energy-containing beverages: Reproductive hormones and ovarian function in the BioCycle Study. Am. J. Clin. Nutr..

[20] Mumford S.L., Dasharathy S.S., Pollack A.Z., Perkins N.J., Mattison D.R., Cole S.R., Wactawski-Wende J., Schisterman E.F. (2013). Serum uric acid in relation to endogenous reproductive hormones during the menstrual cycle: Findings from the BioCycle study. Hum. Reprod..

[21] National Center for Biotechnology Information PubChem Compound Database; CID=15606. https://pubchem.ncbi.nlm.nih.gov/compound/methyl_nonanoate.

[22] Hay N. (2016). Reprogramming glucose metabolism in cancer: Can it be exploited for cancer therapy?. Nat. Rev. Cancer.

[23] Malik V.S., Popkin B.M., Bray G.A., Després J.-P., Willett W.C., Hu F.B. (2010). Sugar Sweetened Beverages and Risk of Metabolic Syndrome and Type 2 Diabetes: A Meta-analysis. Diabetes Care.

[24] National Center for Biotechnology Information 2-Piperidone. https://pubchem.ncbi.nlm.nih.gov/compound/2-Piperidone#section=Top.

[25] Yuan D.L., Liang Y.Z., Yi L.Z., Xu Q.S., Kvalheim O.M. (2008). Uncorrelated linear discriminant analysis (ULDA): A powerful tool for exploration of metabolomics data. Chemom. Intell. Lab..

[26] Mai M., Tönjes A., Kovacs P., Stumvoll M., Fiedler G.M., Leichtle A.B. (2013). Serum levels of acylcarnitines are altered in prediabetic conditions. PLoS ONE.

[27] Ramos-Roman M.A., Sweetman L., Valdez M.J., Parks E.J. (2012). Postprandial changes in plasma acylcarnitine concentrations as markers of fatty acid flux in overweight and obesity. Metab. Clin. Exp..

[28] Stanhope K.L., Havel P.J. (2008). Endocrine and metabolic effects of consuming beverages sweetened with fructose, glucose, sucrose, or high-fructose corn syrup. Am. J. Clin. Nutr..

[29] Perng W., Gillman M.W., Fleisch A.F., Michalek R.D., Watkins S.M., Isganaitis E., Patti M.E., Oken E. (2014). Metabolomic profiles and childhood obesity. Obes. (Silver Springmd.).

[30] Butte N.F., Liu Y., Zakeri I.F., Mohney R.P., Mehta N., Voruganti V.S., Goring H., Cole S.A., Comuzzie A.G. (2015). Global metabolomic profiling targeting childhood obesity in the Hispanic population. Am. J. Clin. Nutr..

[31] Hu H., Tellez-Rojo M.M., Bellinger D., Smith D., Ettinger A.S., Lamadrid-Figueroa H., Schwartz J., Schnaas L., Mercado-Garcia A., Hernandez-Avila M. (2006). Fetal lead exposure at each stage of pregnancy as a predictor of infant mental development. Environ. Health Perspect..

[32] Villalpando S., Garcia-Guerra A., Ramirez-Silva C.I., Mejia-Rodriguez F., Matute G., Shamah-Levy T., Rivera J.A. (2003). Iron, zinc and iodide status in Mexican children under 12 years and women 12–49 years of age. A probabilistic national survey. Salud Publica Mex.

[33] Perng W., Fernandez C., Peterson K.E., Zhang Z., Cantoral A., Sanchez B.N., Solano-Gonzalez M., Tellez-Rojo M.M., Baylin A. (2017). Dietary Patterns Exhibit Sex-Specific Associations with Adiposity and Metabolic Risk in a Cross-Sectional Study in Urban Mexican Adolescents. J. Nutr..

[34] Cantoral A., Contreras-Manzano A., Luna-Villa L., Batis C., Roldan-Valadez E.A., Ettinger A.S., Mercado A., Peterson K.E., Tellez-Rojo M.M., Rivera J.A. (2019). Dietary Sources of Fructose and Its Association with Fatty Liver in Mexican Young Adults. Nutrients.

[35] United States Department of Agriculture (USDA), National Agricultural Library (2009). USDA Food Composition Databases.

[36] Willett W.C. (1998). Implications of total energy intake for epidemiologic analyses. Nutr. Epidemiol..

[37] Perng W., Hector E.C., Song P.X.K., Tellez Rojo M.M., Raskind S., Kachman M., Cantoral A., Burant C.F., Peterson K.E. (2017). Metabolomic Determinants of Metabolic Risk in Mexican Adolescents. Obes. (Silver Springmd.).

[38] Lohman T., Roche A., Martorell R. (1988). Anthropometric Standardization Reference Manual.

[39] Boeke C.E., Oken E., Kleinman K.P., Rifas-Shiman S.L., Taveras E.M., Gillman M.W. (2013). Correlations among adiposity measures in school-aged children. BMC Pediatr..

[40] De Onis M., Onyango A.W., Borghi E., Siyam A., Nishida C., Siekmann J. (2007). Development of a WHO growth reference for school-aged children and adolescents. Bull. World Health Organ..

[41] Orphanidou C., McCargar L., Birmingham C.L., Mathieson J., Goldner E. (1994). Accuracy of subcutaneous fat measurement: Comparison of skinfold calipers, ultrasound, and computed tomography. J. Am. Diet. Assoc..

[42] Bonser A.M., Garcia-Webb P. (1984). C-peptide measurement: Methods and clinical utility. Crit. Rev. Clin. Lab. Sci..

[43] Chavarro J.E., Watkins D.J., Afeiche M.C., Zhang Z., Sánchez B.N., Cantonwine D., Mercado-García A., Blank-Goldenberg C., Meeker J.D., Téllez-Rojo M.M. (2017). Validity of Self-Assessed Sexual Maturation Against Physician Assessments and Hormone Levels. J. Pediatrics.

[44] Watkins D.J., Peterson K.E., Ferguson K.K., Mercado-Garcia A., Ortiz M.T., Cantoral A., Meeker J.D., Tellez-Rojo M.M. (2015). Relating phthalate and BPA exposure to metabolism in peripubescence: The role of exposure timing, sex, and puberty. J. Clin. Endocrinol. Metab..

[45] Hernandez B., Gortmaker S., Laird N., GColditz G., Parra S., Peterson K. (2000). Validez y reproducibilidad de un cuestionario de actividad e inactividad física para escolares de la ciudad de México. Salud Publica Mex..

[46] Tibshirani R. (1996). Regression shrinkage and selection via the Lasso. J. R. Stat. Soc. Ser. B Stat. Methodol.

[47] Stone M. (1974). Cross-Validatory Choice and Assessment of Statistical Predictions. J. R. Stat. Soc. Ser. B.

[48] Bach F.R. Bolasso: Model consistent lasso estimation through bootstrap. Proceedings of the 25th International Conference on Machine Learning.

[49] Davis J.N., Le K.A., Walker R.W., Vikman S., Spruijt-Metz D., Weigensberg M.J., Allayee H., Goran M.I. (2010). Increased hepatic fat in overweight Hispanic youth influenced by interaction between genetic variation in PNPLA3 and high dietary carbohydrate and sugar consumption. Am. J. Clin. Nutr..

[50] Sumner L.W., Amberg A., Barrett D., Beale M.H., Beger R., Daykin C.A., Fan T.W.-M., Fiehn O., Goodacre R., Griffin J.L. (2007). Proposed minimum reporting standards for chemical analysis. Metabolomics.

