# Supplementary material for: Urate and Nonanoate Mark the Relationship between Sugar-Sweetened Beverage Intake and Blood Pressure in Adolescent Girls: A Metabolomics Analysis in the ELEMENT Cohort
Source: Metabolites. 2019 May 17;9(5):100. doi: 10.3390/metabo9050100 (PMC6572261; doi:10.3390/metabo9050100)
Supplement: Supplementary file 1 [file metabolites-09-00100-s001.pdf]

Table S1 Descriptive statistics of age, metabolic outcomes, and pubertal status for 242 ELEMENT participants.

	Girls<br>*n* = 128	Boys<br>*n* = 114	*P*-value [a]
Age, years	10.2 ± 1.7	10.4 ± 1.6	0.39
Glycemia			
Fasting glucose (mg/dL)	86.3 ± 10.5	88.1 ± 7.8	0.13
Fasting C-peptide (ng/mL)	1.8 ± 1.3	1.6 ± 1.2	0.11
CP-IR[b]	0.41 ± 0.38	0.35 ± 0.26	0.17
Leptin (ng/mL)	13.9 ± 10.2	8.3 ± 6.5	<0.0001
Lipid profile			
Total cholesterol (mg/dL)	159.3 ± 27.9	151.5 ± 28.8	0.05
HDL (mg/dL)	57.7 ± 11.8	59.6 ± 12.1	0.25
LDL (mg/dL)	82.1 ± 22.4	76.3 ± 24.0	0.08
Triglycerides (mg/dL)	97.5 ± 47.8	77.7 ± 38.3	0.002
Adiposity and blood pressure			
BMI z-score[c]	0.82 ± 1.31	0.88 ± 1.19	0.63
Waist circumference (cm)	71.5 ± 11.4	69.9 ± 10.0	0.35
SS+TR (mm)	30.5 ± 12.1	25.4 ± 11.0	0.002
SBP (mmHg)	102 ± 10	105 ± 10	0.02
DBP (mmHg)	66 ± 7	66 ± 7	0.71
Indicators of pubertal development			
Pubic hair, Tanner stage >1	24.2% (31)	18.0% (20)	0.37
Breast, Tanner stage >1	32.8% (42)	--	
Testicles, Tanner stage >1	--	50.0% (55)	

[a] *P*-values are from an independent T-test for all variables except for pubic hair, which is from a Likelihood Ratio Chi-squared test.
